# Spin Polarization and Magnetic Moment in Silicon Carbide Grown by the Method of Coordinated Substitution of Atoms

**DOI:** 10.3390/ma14195579

**Published:** 2021-09-26

**Authors:** Sergey A. Kukushkin, Andrey V. Osipov

**Affiliations:** Institute for Problems in Mechanical Engineering of the Russian Academy of Sciences, 199178 Saint-Petersburg, Russia; andrey.v.osipov@gmail.com

**Keywords:** silicon carbide, spin polarization, half-metallic ferromagnet, solid-state spin, density-functional theory

## Abstract

In the present work, a new method for obtaining silicon carbide of the cubic polytype 3C-SiC with silicon vacancies in a stable state is proposed theoretically and implemented experimentally. The idea of the method is that the silicon vacancies are first created by high-temperature annealing in a silicon substrate Si(111) doped with boron B, and only then is this silicon converted into 3C-SiC(111), due to a chemical reaction with carbon monoxide CO. A part of the silicon vacancies that have bypassed “chemical selection” during this transformation get into the SiC. As the process of SiC synthesis proceeds at temperatures of ~1350 °C, thermal fluctuations in the SiC force the carbon atom C adjacent to the vacancy to jump to its place. In this case, an almost flat cluster of four C atoms and an additional void right under it are formed. This stable state of the vacancy, by analogy with NV centers in diamond, is designated as a C_4_V center. The C_4_V centers in the grown 3C-SiC were detected experimentally by Raman spectroscopy and spectroscopic ellipsometry. Calculations performed by methods of density-functional theory have revealed that the C_4_V centers have a magnetic moment equal to the Bohr magneton μB and lead to spin polarization in the SiC if the concentration of C_4_V centers is sufficiently high.

## 1. Introduction

In recent years, much attention has been paid to the creation of solid-state devices, the operations of which are based not on the charge but the spin of charged particles; therefore, the development of new materials with the property of spin polarization is a very important task [1,2,3]. In addition, it is desirable that these materials combine well with the standard materials of microelectronics, such as silicon Si, silicon carbide SiC, or III-V semiconductor compounds. This requirement is very important for scaling and ensuring the relationship between qubits [4]. The most studied solid-body spin is the negatively charged nitrogen-vacancy center (NV) in diamond [1]. In recent years, similar NV centers in SiC [5,6], as well as some other point defects based on silicon vacancies (V_Si_) in SiC [4,7], have been intensely studied.

The key problem in the development of new materials for spintronics based on V_Si_ in SiC is the difficulty in obtaining a sufficient number of silicon vacancies. The work of the V_Si_ formation in silicon carbide of the cubic polytype 3C-SiC is very large and equal to ~8 eV [8], while the work of formation of a carbon vacancy V_C_ in 3C-SiC is much less and equal to ~3.5 eV [9]. This means that it is impossible to obtain V_Si_ in SiC in a thermal way, where only V_C_ vacancies are formed. Therefore, at present, various methods of irradiating SiC with high-energy beams of electrons, ions, or protons are used to create silicon vacancies [10]. The irradiation methods have quite a few disadvantages; for example, significant inhomogeneity of the structure of defects, both in the direction of the beam and perpendicular to it; a large number of undesirable defects (apart from V_Si_); the difficulty of processing large-area samples; etc.

In this work, a fundamentally new method for obtaining silicon vacancies in SiC is proposed. It consists in the fact that the vacancies are initially created not in the SiC, but in the Si, which is much simpler because the energy of vacancy formation in Si is ~3.3 eV [11]. Only then does Si transform into SiC by the Method of Coordinated Substitution of Atoms (MCSA) due to a chemical reaction [12]

2Si (crystal) + CO (gas) = SiC (crystal) + SiO (gas) ↑(1)

In this reaction, part of the silicon vacancies in the Si simply become silicon vacancies in the SiC. Some of the silicon vacancies presumably disappear when Si “collapses” into SiC with a twofold total decrease in the volume of the material. [12]. The term “coordinated” means that the old Si–Si chemical bonds in the silicon are destroyed simultaneously with the formation of new Si–C chemical bonds [13]. The very structure of the bonds does not change in this case. This is what makes it possible to obtain high-quality SiC layers by this method [13]. As the initial bond structure in Si is cubic, it remains the same in SiC, i.e., the cubic 3C-SiC polytype grows (with very few exceptions).

In this work, layers of epitaxial 3C-SiC with a thickness of ~0.1 ÷ 1 μm and a lack of ~0.5 ÷ 5% of Si atoms were produced by the MCSA on 3-inch Si(111) substrates doped with boron B. As the synthesis temperature is high enough (~1350 °C), the vacancies transit into a stable state with a solid-state spin. The produced layers were investigated by spectral ellipsometry (SE), X-ray diffraction (XRD), reflected high-energy electron diffraction (RHEED), and Raman spectroscopy (RS). The magnetic and electrical properties of such a 3C-SiC layer were modeled by methods of the density functional theory (DFT). In particular, it was shown that this layer, in contrast to ideal SiC, has a magnetic moment and the property of spin polarization.

## 2. Materials and Methods

**Figure 1:**
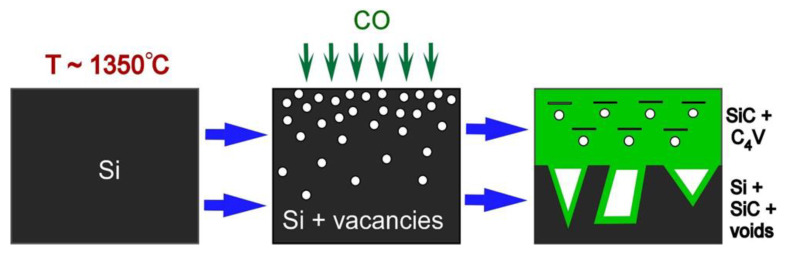
Schematic representation of the transformation of silicon with vacancies into silicon carbide with silicon vacancies. In the case of intrinsic SiC or p-type SiC at high temperatures, metastable silicon vacancies transform into a stable configuration with a solid-state spin: an almost flat cluster of 4 carbon atoms and, under it, a void denoted as C_4_V.

Before the synthesis of the SiC epitaxial layer, 3-inch Si(111) substrates doped with B were purified from oxides in a mixture of NH_4_OH and NH_4_F, which additionally ensured the passivation of the Si(111) surface with hydrogen atoms [14]. Then, the Si substrates were annealed in a vacuum at a temperature *T*~1350–1380 °C (the silicon melting temperature is ~1410 °C) for *t_a_* = 1 ÷ 40 min. Depending on the annealing time, vacancies with different concentrations and penetration depths were formed in the Si surface layer. The energy of formation of a vacancy in the bulk of silicon is ~3.3 eV [11]; however, on a real Si(111) surface, there are steps and kinks, which can significantly reduce the energy of vacancy formation. In addition, we noticed that the emergence of a dislocation on the surface greatly increases the rate of formation of the vacancies. On average, the penetration depth of vacancies deep into the Si(111) substrate at annealing times *t_a_* = 1 ÷ 40 min was 0.5 ÷ 5 μm. The vacancies obtained in this way play a very important role in the synthesis of SiC by the MCSA as the diffusion of both CO into the substrate and SiO out of it proceeds primarily due to vacancies in the reaction (1). After that, carbon monoxide CO was supplied to the furnace, and the epitaxial growth of SiC on Si(111) was carried out according to the approved method [12,13]. The synthesis time was *t_s_* = 10 ÷ 20 min at the CO pressure *p_CO_* = 80 ÷ 200 Pa and the same temperature as during the annealing. The process of SiC growth from Si is schematically shown in Figure 1. It turned out that the preliminary annealing of Si leads to a strong increase in the thickness of the epitaxial SiC layer [15]. In particular, at *T* = 1350 °C, *t_s_* = 15 min, and *p_CO_* = 150 Pa, the measurement of the thickness H_SiC_ of the SiC layer gives the following results: H_SiC_ = 80 nm at *t_a_* = 0, i.e., in the absence of annealing; H_SiC_ = 180 nm at *t_a_* = 2 min; H_SiC_ = 280 nm at *t_a_* = 5 min; and finally, H_SiC_~600 nm at *t_a_* = 25 min, and the latter SiC layer already begins to delaminate from the substrate after cooling the sample to room temperature.

## 3. Results

**Figure 2:**
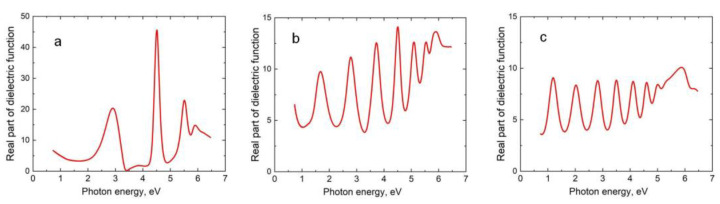
Dependences of the real part of the dielectric constant ε_1_ on the photon energy E for three 3C-SiC samples grown on Si(111) substrate doped with B produced at different times of the preliminary Si annealing: (**a**)—*t_a_* = 0 min, (**b**)—*t_a_* = 2 min, and (**c**)—*t_a_* = 5 min.

The obtained 3C-SiC samples on the Si(111) substrates doped with B (i.e., p-type) were examined by SE, XRD, RHEED, and RS. Figure 2 shows the dependences of the real part of the dielectric constant ε_1_ on the photon energy *E* (measured by the ellipsometer M-2000D J.A. Woollam, USA with a rotating compensator) for three samples grown under the same conditions: *T* = 1350 °C, *P_CO_* = 150 Pa, and *t_s_* = 15 min—but at different times *t_a_* of the preliminary annealing.

**Figure 3:**
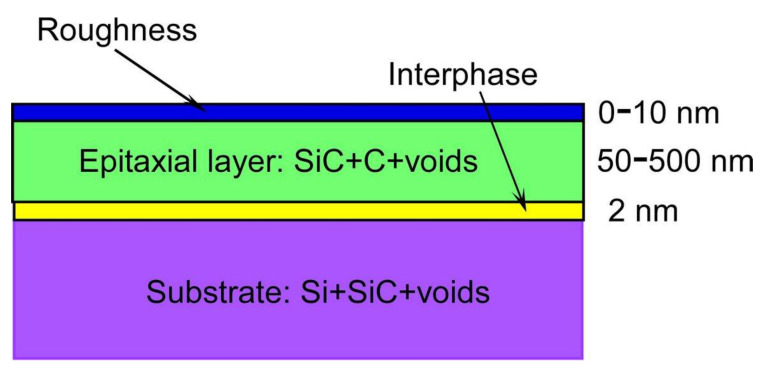
Ellipsometric model used in the processing of the experimental spectra of 3C-SiC/Si(111) samples.

To analyze these dependences, we used a two-layer ellipsometric model (Figure 3) developed in [16] specifically for such samples. The bottom-most layer of the model is the substrate represented by the Bruggeman effective medium approximation (EMA) [16] for the three materials, namely Si, SiC, and voids. The first layer is an interface between the substrate and the main epitaxial SiC layer.

As discovered in [16], it has a low conductivity due to the breaking of the bonds of some Si atoms of the substrate, which are attracted to the SiC. Hence, the dielectric constant of this layer, the thickness of which is almost constant and equal to 2 nm, is best described by the Tauc-Lorentz (TC) model [16]. The second layer is an epitaxial SiC layer, the dielectric constant of which is described by the Bruggeman EMA [16] for a mixture of three materials: SiC, crystalline graphite C, and voids—where the volume concentration of C and the voids is considered the same. It was shown earlier in [17] that this EMA model very well describes the SiC with silicon vacancies in a stable state. On top of the second layer, there is a standard roughness layer. Minimization of the difference between the measured dielectric constant and the one calculated within the framework of this two-layer ellipsometric model yields the values of the model parameters, including the thickness of the SiC layer and the volume concentration of C and the voids in it. Of course, the SiC thickness determined by SE is exactly the same as that obtained from SEM pictures. It should be emphasized that the SE method is effective only for the examination of SiC layers with a thickness less than 400 nm. If the SiC thickness is more than 400 nm, then, first, due to an increase in the concentration of silicon vacancies, the SiC ceases to be transparent and gradually turns into a conductor (more specifically, into a half-metallic ferromagnet). As a result, the laser beam of the ellipsometer is not reflected from the bottom of the SiC layer, so the interference that leads to a large number of peaks of the dielectric constant (Figure 2) disappears. This makes it impossible to use the described ellipsometric model. Second, when the thickness of the SiC is more than 0.5 μm, it begins to delaminate from the substrate, which also leads to the destruction of the interface between the SiC and the Si and to the disappearance of the interference. Hence, for the thick SiC layers, only an SEM has to be used to determine the thickness.

**Figure 4:**
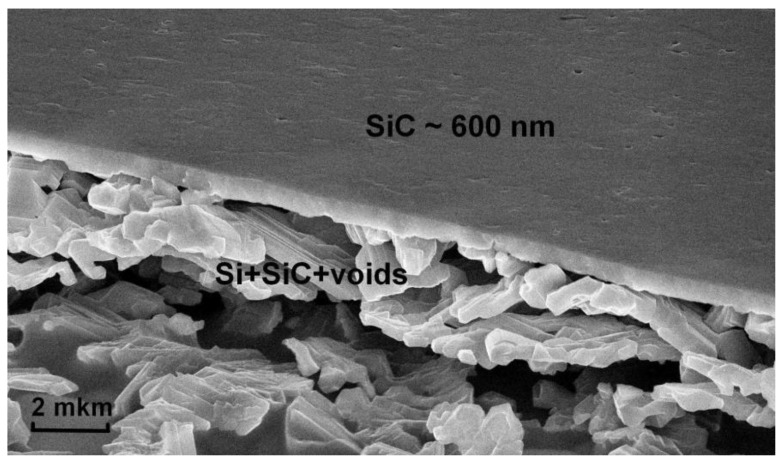
SEM image of a section of a 3C-SiC/Si(111) sample produced at *t_a_* = 25 min and starting to delaminate from the substrate.

Figure 4 shows an SEM image of a section of a 3C-SiC/Si(111) sample with an epitaxial SiC layer of ~600 nm thickness, produced at *t_a_* = 25 min, *T* = 1350 °C, *P_CO_* = 150 Pa, and *t_s_* = 15 min. Under the SiC layer, there is a mixture of Si, SiC, and voids formed due to the fact that the volume of the SiC cubic cell is half the volume of the original Si cell. The thickness of this mixture (it is ~5 μm for the given sample) strongly depends on the time of preliminary annealing of the substrate and is in the range of 0.5 ÷ 5 μm. We suppose that it is approximately equal to the penetration depth of the vacancies upon the Si annealing.

**Figure 5:**
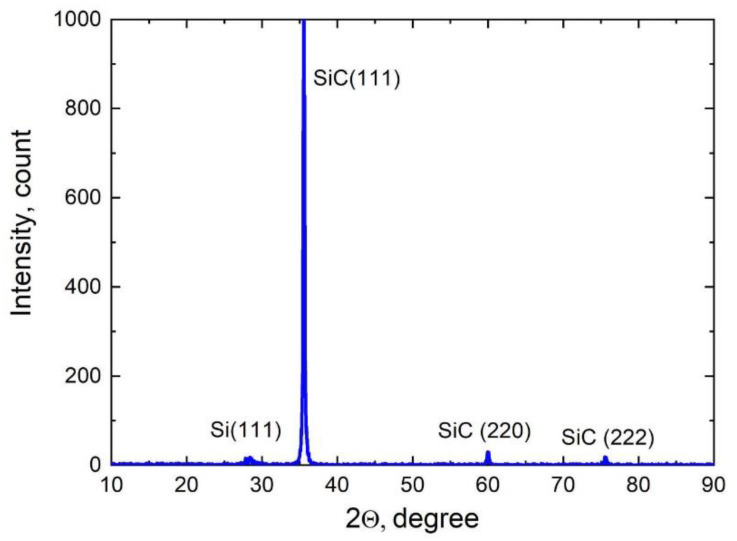
XRD pattern of the 3C-SiC/Si(111) sample produced at *t_a_* = 25 min.

Figure 5 shows the XRD spectrum obtained by Dron-8, Burevestnik, Russia of the same sample as in Figure 4. This sample has already started to delaminate from the substrate; hence, the peak corresponding to the Si(111) substrate is very small. If the SiC had not yet delaminated from the Si, then the signal amplitude would be about 1000 times greater. The half-width of the main 3C-SiC(111) peak is 6 arcmin, which indicates the epitaxial quality of the layer. In addition to the peaks of the main orientation (111) and (222), there is a very small peak of the orientation (220). It should be taken into account that the XRD spectrum in Figure 5 concerns not only the 600-nm-thick 3C-SiC(111) epitaxial layer but also the entire porous SiC layer under it, which is of inferior quality and contains 3C-SiC grains of various orientations.

**Figure 6:**
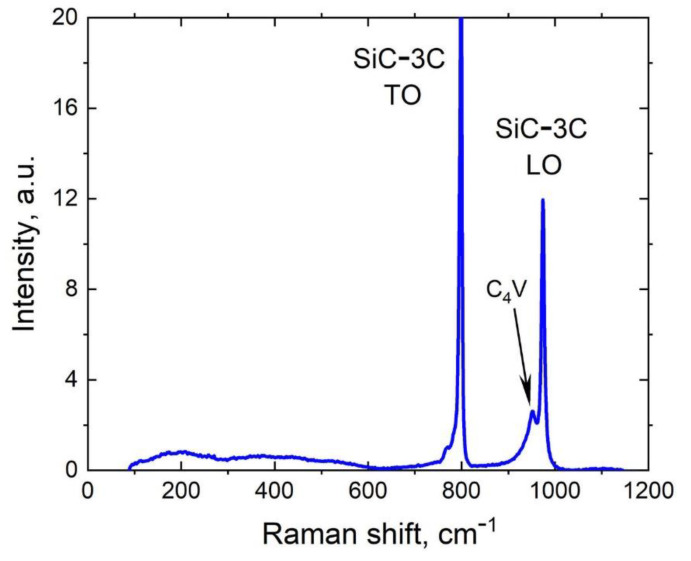
RS of the 3C-SiC/Si(111) sample produced at *t_a_* = 25 min. There is an additional peak at 952 cm^−1^, corresponding to silicon vacancies in a stable state (C_4_V).

The Raman spectrum of the same sample obtained by confocal raman microscope WiTec Alpha300R, Ulm, Germany is shown in Figure 6. As the sample has already started to delaminate, there is no silicon line. In addition to the two peaks TO and LO of the 3C-SiC, there is an additional peak at 952 cm^−1^ which we associate with the silicon vacancies that have transited to a stable state denoted as C_4_V. The DFT modeling has shown that it is exactly the oscillation frequency of the C–C bonds arising in the 3C-SiC when a C atom jumps over to the place of the silicon vacancy [18]. Previously, by different research teams, this line was repeatedly observed in the infrared spectra (both transmission and reflection, which is much more sensitive than the Raman spectroscopy) of the 3C-SiC samples produced by the MCSA but with a significantly lower concentration of vacancies [19].

**Figure 7:**
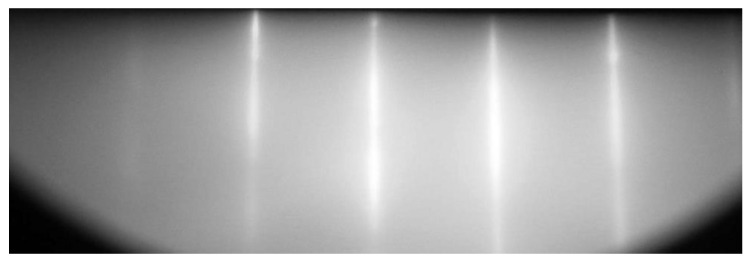
RHEED pattern of the 3C-SiC/Si(111) sample produced at *t_a_* = 25 min.

Figure 7 shows the RHEED pattern obtained by the EMR-100 (Ukraine) electron-diffraction instrument with the energy of electrons of 50 keV for the same sample of ~600 nm thickness after its cooling. For the given energy of electrons, the depth of their penetration into the sample does not exceed ~100 nm, so this diffraction pattern corresponds only to the upper layer of the epitaxial SiC. It is clear that the diffraction pattern corresponds to a smooth epitaxial layer. There are absolutely no patterns corresponding to a polycrystalline or amorphous structure.

So, after the preliminary annealing of the Si, a number of silicon vacancies are formed in its surface layer. Then, as a result of the chemical substitution reaction (1), the Si “collapses” to 3C-SiC with a twofold decrease in the volume of the material (the volume of the Si cubic cell with eight atoms is 160 Å^3^, whereas the volume of the 3C-SiC cubic cell with eight atoms is 83 Å^3^). The “collapse” in the uppermost layer occurs vertically, i.e., the lateral size remains almost unchanged, which provides an even and uniform SiC layer (Figure 4) [13]. In the lower layers, the “collapse” occurs in all directions, resulting in a porous layer under the SiC epitaxial film, consisting of SiC, Si, and voids (Figure 4). The voids arise precisely because the volume of SiC is approximately two times less than the volume of the initial Si. The vacancies in the original Si allow CO and SiO to diffuse first into the Si and then into the SiC, which results in a much thicker SiC layer. In principle, a 3C-SiC layer with a thickness of more than 1 μm can be obtained at a maximum temperature of 1380 °C and an annealing time of *t_a_*~40 min, whereas it is impossible to obtain a layer thicker than 100 nm without preliminary annealing.

Apparently, some part of the silicon vacancies upon “collapse” simply transits from the Si to the SiC, i.e., these vacancies are of a purely chemical origin. Moreover, elastic stress is an additional source of V_Si_ in SiC due to ascending diffusion [20]. Therefore, the SiC produced by the MCSA inevitably contains V_Si_. The minimum energy corresponds here to the periodic distribution of the vacancies due to their repulsion. It can even be said that in this technique, unlike in the other techniques for growing SiC, it is impossible to get rid of the V_Si_. The only question is what their concentration is. Obviously, the preliminary annealing of the Si significantly increases the concentration of V_Si_ in the 3C-SiC. SE allows the measurement of the volumetric concentration of V_Si_ by the height and shape of the interference peaks of the dielectric constant (Figure 2). The lower the peaks, the higher the concentration of V_Si_. The SE method gives the volume concentration of V_Si_ approximately in the range of 0 ÷ 5% after annealing for *t_a_* = 0 ÷ 30 min at *T* = 1350 °C. It should be emphasized that the accuracy of the SE method is not high because there is a strong correlation between the concentration of the vacancies and the depolarization parameter in the Bruggeman method [16].

## 4. Modeling of the Magnetic and Electrical Properties of 3C-SiC Produced by the Method of Coordinated Substitution of Atoms (MCSA)

In this work, we use the DFT methods implemented in the Medea-VASP software [21] to model the magnetic and electrical properties of the 3C-SiC produced by the MCSA with the preliminary annealing of Si. In all our computations for the systems under study, periodic boundary conditions are used, as well as the spin-polarized approach [22] allowing us to adequately describe the magnetic moment in the 3C-SiC. The exchange-correlation interaction is calculated here using the PBE functional [23]. For integration over the Brillouin zone, we use the Monkhorst-Pack grid of k-points spaced apart by less than 0.17 Å^−1^. In all our calculations, pseudopotentials using the projector augmented-wave (PAW) method [21] are utilized; the cutoff energy of the waves is taken as equal to 400 eV. The band structure and the density of the electronic states are calculated here using the MBJLDA meta-GGA functional [24], which provides a very high accuracy in calculating the bandgap and, moreover, adequately describes the magnetic moment.

**Figure 8:**
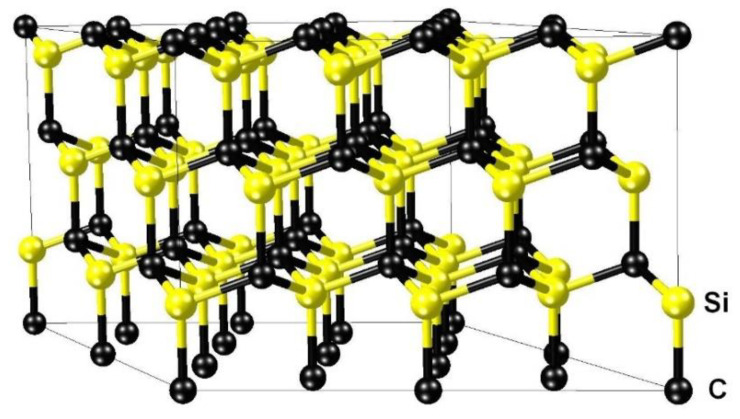
The model system containing 36 Si atoms and 36 C atoms with periodic boundary conditions in all three dimensions and initial size parameters *a* = *b* = 10.68 Å, *α* = 60 °, and *c* = 7.55 Å.

First of all, it is necessary to compute the configuration of the 3C-SiC with the V_Si_, corresponding to the minimum energy, and to calculate its magnetic moment. It is known that if a Si atom is simply removed from SiC, either p-type or compensated, then such a vacancy corresponds to a metastable state because it is favorable for one of the four C atoms closest to the V_Si_ vacancy to jump over to the place of the V_Si_ [9,18]. In n-type SiC, such a jump may be disadvantageous [9]. Let us calculate this process by taking into account the magnetic moment. As a model system, we consider the 3C-SiC supercell consisting of 36 Si atoms and 36 C atoms, where the <111> direction in the SiC is oriented upward along the Z-axis (Figure 8). The removal of any Si atom in such a system leads to a decrease in symmetry from *F*$\bar{4}$3*m* to *P*31*m*. The jump of an adjacent C atom from underneath to the place of the absent atom along the Z-axis retains the *P*31*m* symmetry.

**Figure 9:**
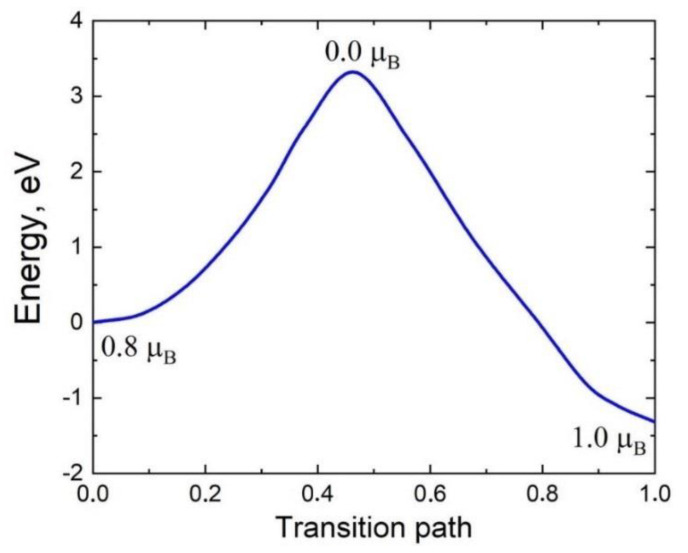
Energy profile of the transition of a silicon vacancy in the model system (Figure 8) from the initial metastable state V_Si_ to stable C_4_V. The transition proceeds through a nonmagnetic transition state.

Such a displacement of an atom is best described by the Nudged Elastic Band (NEB) method [25]. The idea of this method is that knowing the initial and final states, one can freeze the system and introduce additional forces that will direct the atoms from the initial state to the final state. If certain conditions are met, the system will follow the Minimal Energy Pathway (MEP) [25]. This is a transition pathway where any local change in the path leads to an increase in the energy of the system in the vicinity of a given point of the pathway. The advantage of the NEB method is that if the initial and final configurations of the system (which are obtained by minimizing the electron energy) are known, then all the intermediate and transition states are found [25]. An application of the NEB method in the spin-polarized approximation to the jump of a C atom over to the place of the V_Si_ gives the energy profile of this process. The calculation results are shown in Figure 9.

Thus, the overjump of a C atom gives a gain of 1.3 eV, i.e., the vacancy from the initial metastable state V_Si_ transits to the stable state C_4_V. The key point here is the fact that the initial and final states have a nonzero magnetic moment (in the PBE approximation, *μ* = 0.8 μB and *μ* = 1.0 μB, respectively; μB is the Bohr magneton), whereas the transition state corresponding to the maximum energy is nonmagnetic (*μ* = 0). The energy barrier height is 3.33 eV, which is 10% more than the corresponding result obtained without considering the magnetic moment. The value of 3.33 eV means that at a temperature *T* > 1200 °C all vacancies must pass to a stable state in a time less than 1 s. So, in the MCSA, all vacancies must be in a stable state.

**Figure 10:**
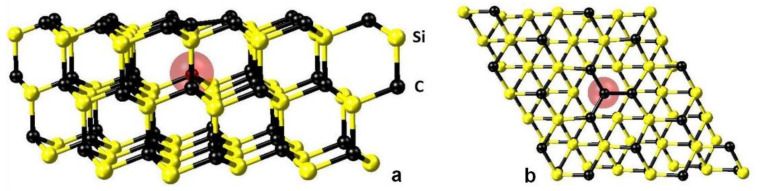
The stable state of a C_4_V silicon vacancy in p-type 3C-SiC (or intrinsic), consisting of an almost flat cluster of 4 C atoms and a void shown as a semi-transparent red sphere. (**a**)—view in the direction <0$\bar{1}$1>, (**b**)—view in the direction <100>.

The stable state of a silicon vacancy in 3C-SiC, corresponding to the minimum energy, is shown in Figure 10. The C atom, after jumping over to the place of the V_Si_, forms an almost flat cluster of four C atoms with a bond length of 1.57 Å. The calculations show that the wavenumber for these bonds is approximately equal to 970 cm^−1^ [18]. This is why we believe that the new peak of the Raman spectrum at 952 cm^−1^ (Figure 6) is associated with the vibrations of the C-C bonds. A similar line was also found in the DFT calculations in the 3C-SiC containing carbon antisites [26]. Because of the described overjump, an additional void with a diameter of 1.85 Å appears under the carbon cluster at a distance of 2.0 Å. The void is shown in Figure 10 as a semi-transparent red sphere. This void is inextricably linked with the carbon cluster and affects the electrical and magnetic properties of the system. By analogy with NV centers in diamond, we call this formation a C_4_V center. It is important to note that the carbon vacancy located under the C_4_ carbon cluster strongly interacts with it, providing a magnetic moment to it. In order for this carbon vacancy to diffuse to another place, an energy of 1.2 eV must be expended. In this case, the carbon cluster ceases to be flat and completely loses its magnetic moment. Thus, in the absence of additional electric charges the weakly magnetic metastable state V_Si_ transforms into a magnetic stable state C_4_V at a temperature *T* > 1200 °C through a nonmagnetic transition state (Figure 8). The calculations show that the magnetic moment of the C_4_V center is equal to the Bohr magneton, i.e., *μ* = 1.0 μB.

**Figure 11:**
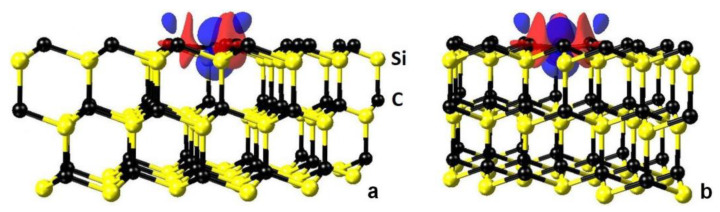
The difference in the density of electrons with spin up and spin down in the model system with a C_4_V center. The border of the blue region corresponds to a difference of 0.05 Å^−1^; the border of the red region corresponds to a difference of −0.01 Å^−1^. (**a**) and (**b**)—views from different sides.

**Figure 12:**
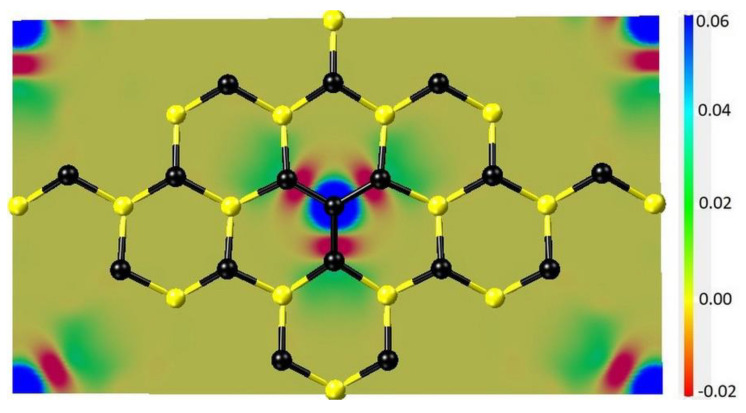
Projection of the difference in the density of spin-up and spin-down electrons onto the (100) plane passing through the centers of Si atoms just below the carbon cluster C_4_. The color scale spans from −0.02 Å^−1^ (red) to 0.06 Å^−1^ (blue).

In ordinary nonmagnetic systems, including ideal SiC, the density of spin-up electrons at each point in the system is equal to the density of spin-down electrons. The situation is different with the SiC produced by the MCSA, i.e., containing C_4_V centers. The reason is that the central C atom in the cluster has one unpaired p-electron (thus, C_4_V centers in SiC are in many ways similar to the NV centers in diamond). It is this electron that radically changes the electrical and magnetic properties of SiC. Figure 11 shows the difference in the density of spin-up and spin-down electrons in the model system with a C_4_V center. The border of the blue region corresponds to a difference of 0.05 Å^−1^ and the border of the red region corresponds to a difference of −0.01 Å^−1^. The central blue region, looking like a three-dimensional figure of eight, corresponds to the position of the unpaired p-electron of the C atom located in the center of the C_4_ cluster. The total value of the magnetic moment of one C_4_V center is 1 μB. Figure 12 shows the projection of the difference in the density of spin-up and spin-down electrons onto the (100) plane passing through the centers of the Si atoms just below the carbon cluster C_4_.

**Figure 13:**
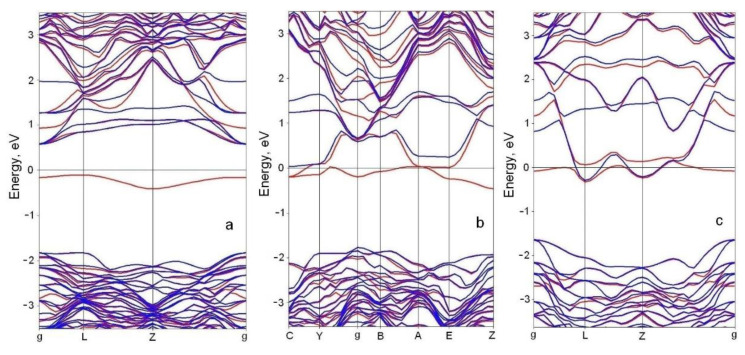
Band structure of three systems with different concentrations *n*_C_4_V_ of defects. (**a**)—system I, $n_{C_{4}V}$ = 2.8%; (**b**)—system II, $n_{C_{4}V}$ = 4.2%; (**c**)—system III, $n_{C_{4}V}$ = 6.3%. The red curves correspond to electrons with spin up, the blue curves to those with spin down.

The presence of stable silicon vacancies, i.e., C_4_V centers, transforms SiC into a system with a solid-state spin, which radically changes not only the magnetic but also the electrical properties of SiC. The band structure of 3C-SiC strongly depends on the concentration of C_4_V centers and on their mutual arrangement, i.e., on the symmetry group. In this case, a new band structure is formed, and the substance, while remaining silicon carbide on the chemical level, turns into a substance with completely different electrophysical and magnetic properties. Calculations describing the effect of point defects on the band structure often take into account the effect of an electric charge localized near the defects. For p-type conductivity, the most energetically favorable charge is +e [9]. Therefore, in the present work, this particular case is investigated. Three systems with different concentrations of C_4_V centers are considered. System I (Figure 10) has the symmetry group *P*31*m* and the dimensions *a* = *b* = 10.60 Å, *α* = 60°, and *c* = 7.58 Å; it contains 35 Si atoms and 36 C atoms with the concentration of C_4_V centers being $n_{C_{4}V}$ = 1/36 ≈ 2.8%, i.e., every 36th Si atom is absent. System II has the symmetry group *Pm* and the dimensions *a* = *b* = 10.52 Å, *α* = 90 °, and *c* = 6.10 Å; it contains 23 Si atoms and 24 C atoms with $n_{C_{4}V}$ = 1/24 ≈ 4.2%, i.e., every 24th Si atom is absent. System III has the symmetry group *P*3*m*1 and the dimensions *a* = *b* = 6.04 Å, *α* = 60 °, and *c* = 10.11 Å; it contains 15 Si atoms and 16 C atoms with $n_{C_{4}V}$ = 1/16 ≈ 6.3%. The results of the computations of the band structures of these three systems are shown in Figure 13. The red curves correspond to electrons with spin up; the blue curves correspond to electrons with spin down.

**Figure 14:**
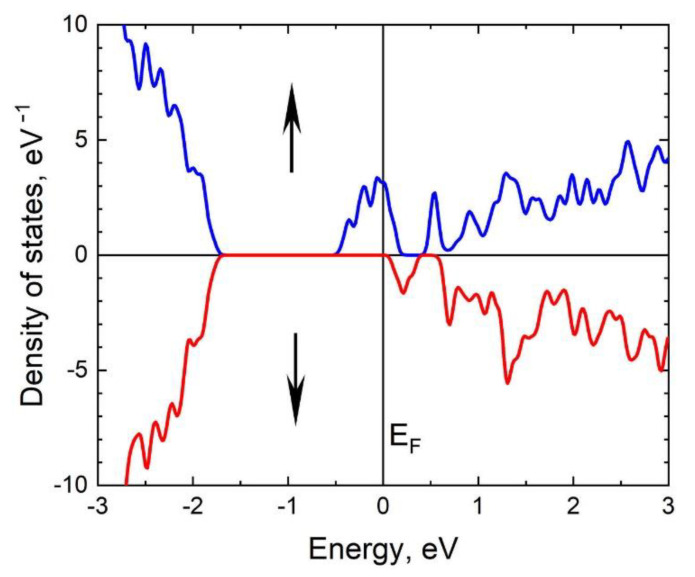
Density of spin-up and spin-down electronic states for system II with $n_{C_{4}V}$ ≈ 4.2%. The blue curve in the upper half describes the spin-up electrons, the red curve in the lower half describes the spin-down electron. The system is a half-metallic ferromagnet with 100% spin polarization.

One can see that the C_4_V centers make a different band structure for electrons with different spins. At a low concentration of C_4_V centers, the 3C-SiC becomes a ferromagnetic semiconductor; its bandgap depends on the spin. For electrons with spin down, the band structure almost does not change compared to the ideal 3C-SiC; the bandgap remains equal to 2.4 eV. On the other hand, for electrons with spin up, the bandgap decreases to 0.6 eV (Figure 13a). With an increase in the concentration $n_{C_{4}V}$, this difference increases, leading to qualitative changes. In particular, system II is already a metal for electrons with spin up, while for electrons with spin down, system II remains a semiconductor with an indirect band of 1.8 eV (Figure 13b). So, the 3C-SiC grown on a silicon substrate by the MCSA is a half-metallic ferromagnet with 100% spin polarization. The density of spin-up and spin-down electronic states for system II is shown in Figure 14.

**Figure 15:**
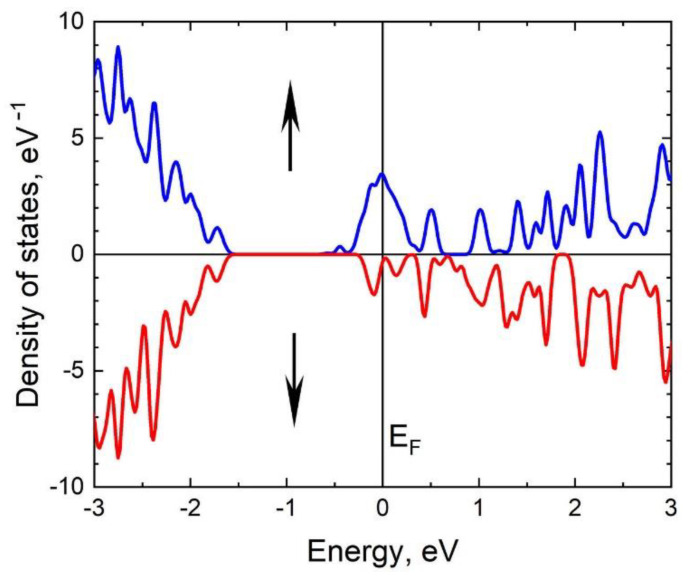
Density of spin-up and spin-down electronic states for system III with $n_{C_{4}V}$ ≈ 6.3%. The blue curve in the upper half describes the spin-up electrons; the red curve in the lower half describes the spin-down electrons. The system is a ferromagnetic metal with a spin polarization of 84%.

With a further increase in $n_{C_{4}V}$, the 3C-SiC turns into a magnetic metal (system III, Figure 13c), however, the concentration of free electrons (i.e., at the Fermi level) with spin up significantly exceeds the concentration of free electrons with spin down, which provides a high value of spin polarization in this case as well. In particular, for system III, the spin polarization is 84%. The density of spin-up and spin-down electronic states for system III is shown in Figure 15.

## 5. Conclusions

In the present work, a new method is theoretically proposed and experimentally implemented for obtaining epitaxial 3C-SiC layers containing silicon vacancies in a stable state with a solid-state spin. The idea of the method is that silicon vacancies are preliminarily created by the annealing of Si, and only then is this Si transformed into SiC by the MCSA due to chemical reaction (1) (Figure 1). In this process, some of the silicon vacancies transit from the Si to the SiC. This chemical mechanism of vacancy generation is realized because the energy of formation of silicon vacancies in Si (~3.3 eV) is much lower than the energy of formation of silicon vacancies in SiC (~8 eV). The DFT calculations have revealed that in the case of p-type conductivity at a temperature above 1200 ℃, thermal fluctuations in SiC lead to a jump of a C atom in the <111> direction to the place of V_Si_. This jump from a metastable state with a magnetic moment of *μ* = 0.8 *μ_B_* (in the PBE approximation) to a stable state with *μ* = 1.0 *μ_B_* proceeds through an intermediate nonmagnetic state with *μ* = 0 (Figure 9). The stable state of a silicon vacancy in SiC is an almost flat cluster of four C atoms (with a C-C bond length of 1.57 Å) and an additional void with a diameter of 1.85 Å, located at a distance of 2.0 Å below the cluster (Figure 10). In terms of its properties, the stable state of a silicon vacancy in SiC is similar to the NV center in diamond; so, by analogy, we call it the C_4_V center. In the produced 3-inch 3C-SiC samples (Figure 4), C_4_V centers are recorded both by Raman spectroscopy (line at 952 cm^−1^, Figure 6) and spectroscopic ellipsometry (by a decrease in the amplitude of the peaks of the dielectric constant, Figure 2). The DFT calculations have revealed that the C_4_V centers in the 3C-SiC strongly affect its magnetic and electrical properties. While the ideal 3C-SiC grown by a standard method is a nonmagnetic semiconductor, the 3C-SiC produced by the MCSA, depending on $n_{C_{4}V}$, is a ferromagnetic semiconductor (Figure 13a), a half-metallic ferromagnet (Figure 13b), or a magnetic metal (Figure 13c). We emphasize that the value of $n_{C_{4}V}$ can be controlled in the MCSA by choosing the time and temperature of the vacuum annealing of the Si. The boundaries between different states of 3C-SiC are vague and depend on the relative position of the C_4_V centers, i.e., on the symmetry group, and on the position of the Fermi level, i.e., the doping level of the original Si. The property of spin polarization (Figure 14 and Figure 15) possessed by the 3C-SiC obtained by the MCSA makes this material extremely promising for various applications. In our opinion, the anomalous magnetic properties of 3C-SiC produced by the MCSA, which were experimentally discovered quite recently [27], can be explained by the presence of C_4_V centers.

## Data Availability

Data is contained within the article.
